# What Could Explain the Lower COVID-19 Burden in Africa despite Considerable Circulation of the SARS-CoV-2 Virus?

**DOI:** 10.3390/ijerph18168638

**Published:** 2021-08-16

**Authors:** Richard G. Wamai, Jason L. Hirsch, Wim Van Damme, David Alnwick, Robert C. Bailey, Stephen Hodgins, Uzma Alam, Mamka Anyona

**Affiliations:** 1 Department of Cultures, Societies, and Global Studies, Northeastern University, 201 Renaissance Park, 360 Huntington Ave., Boston, MA 02115, USA; hirsch.ja@northeastern.edu; 2 Department of Public Health, Institute of Tropical Medicine, B-2000 Antwerp, Belgium; WVDamme@itg.be; 3 DUNDEX (Deployable U.N.-Experienced Development Experts), FX68 Belturbet, Ireland; dalnwick@gmail.com; 4 School of Public Health, University of Illinois at Chicago, Chicago, IL 60607, USA; rcbailey@uic.edu; 5 School of Public Health, University of Alberta, Edmonton, AB T6G 1C9, Canada; shodgins@ualberta.ca; 6 Researcher Africa Institute for Health Policy Foundation, Nairobi 020, Kenya; u.alam@aasciences.africa; 7 T.H. Chan School of Public Health, Harvard University, Boston, MA 02115, USA; mamka.anyona@gmail.com

**Keywords:** COVID-19 pandemic, Africa, SARS-CoV-2 virus spread, lower COVID-19 disease burden, African populations, demographic pyramid, trained immunity, government measures

## Abstract

The differential spread and impact of severe acute respiratory syndrome coronavirus 2 (SARS-CoV-2), causing Coronavirus Disease 2019 (COVID-19), across regions is a major focus for researchers and policy makers. Africa has attracted tremendous attention, due to predictions of catastrophic impacts that have not yet materialized. Early in the pandemic, the seemingly low African case count was largely attributed to low testing and case reporting. However, there is reason to consider that many African countries attenuated the spread and impacts early on. Factors explaining low spread include early government community-wide actions, population distribution, social contacts, and ecology of human habitation. While recent data from seroprevalence studies posit more extensive circulation of the virus, continuing low COVID-19 burden may be explained by the demographic pyramid, prevalence of pre-existing conditions, trained immunity, genetics, and broader sociocultural dynamics. Though all these prongs contribute to the observed profile of COVID-19 in Africa, some provide stronger evidence than others. This review is important to expand what is known about the differential impacts of pandemics, enhancing scientific understanding and gearing appropriate public health responses. Furthermore, it highlights potential lessons to draw from Africa for global health on assumptions regarding deadly viral pandemics, given its long experience with infectious diseases.

## 1. Background

As of 11 August 2021, approximately 7.1 million confirmed COVID-19 cases were reported in Africa continentwide [1]. Now, a year and a half since the first infection was reported in Egypt on 14 February 2020, Africa has accounted for just 3.5% of 204.2 million lab-confirmed cases [2], despite containing 12.5% of the global population [3]. Africa’s share of deaths is just 4.1% of the 4.3 million reported globally [2]. These numbers significantly defy early predictions of mass COVID-19 catastrophe [4,5,6,7]. The doom and gloom predictions were based on what was known about how the disease is transmitted, and how socially deprived settings, unsanitary living conditions, and weak health systems, which are common throughout the continent, could exacerbate spread and subsequent disease burden [6,8,9]. To date, mass infection spread, high rates of severe disease, and excess mortality due to COVID-19 on the continent have not been reported [10]. One important exception is South Africa, which carries a substantial 35% of the confirmed cases and 42% of total deaths among the 55 countries on the continent [1]. This leads to questioning the differences in reporting between African countries, as South Africa makes up only 4.8% of the 1.2 billion people living in Africa and has conducted a disproportionate 24% of the 50.6 million tests administered, as of 17 June 2021 [1].

Because of low testing capacities, Africa has conducted the least number of tests of all global regions given its population size [1,11], but has exceeded the Africa Centers for Disease Control and Prevention (Africa CDC) targets of 8000 tests per million [1]. This should be seriously considered as having contributed to an underestimation of cases [12]. Several seroprevalence studies offer insights on the extent of spread in the continent. For example, a cross-sectional household study in Zambia reported much higher infections than reported via the limited normative testing, which showed only one confirmed case reported for every 92 community infections [13]. Small antibody studies among healthcare workers in hospitals have reported up to a 36% prevalence in Kinshasa, DRC [14], 45.1% in Ibadan, Nigeria [15], and 12.3% in Blantyre, Malawi [16], during the period of May to June 2020. Studies among blood donors in Kenya and South Africa from April to June 2020 report anti-SARS-CoV-2-IgG seroprevalence of, respectively, 4.5% nationally [17] and 60% among South African black populations, seven times that of in-country white populations [18]. Two studies reported SARS-CoV-2-IgG positivity of 23.7% in workers of low socio-economic status in Cape Town, South Africa [19], and 25.1% in gold mine workers and administrative staff in Ivory Coast [20]. These studies collected blood samples between April and October 2020 during the first wave of the COVID-19 spread throughout the continent, with the second wave largely peaking around December 2020 [21]. While these studies offer insights, the results are variable. A true picture requires more and larger antibody studies in other geographies and populations over time [22]. Additionally, these data should be interpreted with caution, as none of these tests have been validated in African-specific contexts where it is possible that cross-reactivity with other prevalent viruses, micro-organisms, and hypergammaglobulinemia due to malaria exposure may influence the sensitivity and/or specificity of these tests, potentially leading to either an underestimation or overestimation of seroprevalence [14,23,24,25,26,27].

The integrity of reporting case and death data in Africa has been repeatedly called into question. For instance, in January 2021, *The New York Times* published an article titled “A Continent Where the Dead Are Not Counted” [28]. However, while only 34.6% of countries globally have complete death registration data in the Civil Registration and Vital Statistics (CRVS), most African countries have a system in place [29], and there is no evidence that COVID-19 mortality data is less accurately reported in Africa than elsewhere. Only Tanzania ceased reporting COVID-19 cases or deaths since May 2020 [1]. The World Mortality Dataset reports the undercounting of COVID-19 deaths from many of the 77 countries included in the dataset, such as the U.S., U.K., and Russia, with South Africa, Egypt, and Mauritius being the only African countries listed [30]. While there are some reports of excess deaths from the continent [30,31], these may well be more due to the adverse indirect effects of pandemic prevention measures, such as lockdowns, causing a wide range of difficulties such as food shortages, and a near unilateral focus on COVID-19, diverting resources from treating other diseases and health conditions [32]. Despite having several limitations [33], data from an autopsy study in a small sample of deaths from a teaching hospital in Zambia indicated the underestimation of COVID-19 mortality is a problem in Africa [34]. Nevertheless, rapid mortality assessments for COVID-19 that is underway in some countries [35,36,37,38] could reveal a more complete picture. Expectedly, due to weak health systems, recent data show that Africa has a higher mortality in those with critical COVID-19 illness than elsewhere, with a mortality rate of 48.2% compared with a global average of 31.5% [39].

While various studies have postulated that demographic profile, early actions such as lockdowns, community factors, and possibly population-specific innate immune factors that are yet undetermined [9,12,40] have played a role in the apparently lower COVID-19 burden, the data and speculation still leave many questions unanswered [41,42]. Several articles in the popular media [43,44] postulate hypotheses, but to our knowledge there has not yet been a complete scholarly review. We are beginning to understand that context and history matter a great deal [45,46,47]. The first modelling of the pandemic for nearly all countries in Africa, based on multiple context-specific covariates, has more closely predicted what has been observed [48]. Here, a set of hypotheses to explore observations regarding SARS-CoV-2 spread and a comparatively low COVID-19 disease burden in the African region are examined. An analysis of factors underlying the spread and burden is important because of the potential for valuable global public health lessons, expanding on what is known regarding deadly viral pandemics, population and systems-level preparedness, and subsequent response.

## 2. On SARS-CoV-2 Spread

To understand the full picture of COVID-19 in Africa, we must first examine how spread patterns emerged, and what variables could have influenced these patterns. While recent data have shown extensive SARS-CoV-2 spread in the continent, this was not always thought to be the case, and the extent of the spread compared with other global regions is still somewhat debated. Below, we present three main areas of interest regarding viral propagation in an Africa-specific context.

### 2.1. Early Government Measures and Messaging

Many governments in Africa enacted early response measures to the pandemic [49,50,51,52]. On 5 February 2020, even before there was a single case reported in the continent, the Africa CDC had established the Africa Taskforce for Coronavirus (AFCOR), and on 22 April 2020, the WHO highlighted examples of how Africa was leading the global response. By 15 April 2020, 96% of the 50 African countries examined had in place at least five ‘stringent public health and social measures’ to prepare for the emerging pandemic [21]. Less international connectivity, early border closures, and lockdowns to prevent viral importation from international flight arrivals, especially from China [8], were associated with a lower case load [53]. Modelling studies also found reduced connectivity/travel at regional, national, and international levels as having an important early impact on slowing the spread [48,54].

Destructive epidemics are not new phenomena for Africa. The continent is constantly dealing with abundant infectious disease (e.g., malaria, yellow fever, tuberculosis, Ebola, polio) [55]. Due to their familiarity with these epidemics, many governments have developed effective public health programs with messaging aimed at unifying the community and highlighting the need for preventative action among individuals [56,57]; for example, the case of the response to Ebola in Western Africa [58,59,60] and to the HIV/AIDS epidemic in Uganda [61]. A similar unity around public health messaging has emerged around COVID-19 in many countries, including outside Africa in Vietnam (e.g., “Fighting the epidemic is like fighting against the enemy”) [12]. It is very possible that a certain baseline individual and community preparedness, awareness, and adherence to government public health recommendations on non-pharmaceutical interventions, and a readiness to adapt to a new epidemic had significant implications in stunting disease spread in the community. 

Recent studies point to cultural adherence to government recommendations as being important for mitigating SARS-CoV-2 spread [62,63]. But are Africans, on average, more willing than other populations to respect in-country public health guidelines? Several surveys on adherence to masking, social distancing, and hand hygiene have been conducted in multiple countries, some of which are nationally representative [64]. Studies from Nigeria, Malawi, Ethiopia, Ghana, Kenya, and the DRC showed that although most participants had a good knowledge about COVID-19 transmission modes and prevention mechanisms that were consistent over place and time, there were gaps in the practices that prevent COVID-19 [65,66,67,68,69]. Studies further determined that individuals’ age, sex, educational status, occupation, and income level were associated with COVID-19 related practices [65,67,70,71]. From these studies, it can be concluded that awareness and (non)adherence to NPIs does not explain low reported cases. 

### 2.2. Population Distribution and Structure of Social Networks

Population structure and spatial distribution strongly predict the patterns of SARS-CoV-2 transmission in communities [72,73]. Analysis of spatial and temporal clustering of populations shows a correlation between density/crowding and viral reproduction number [72]. Africa is the least urbanized global region, with 55% of the continent’s population living in rural areas with wide variations across countries [74,75]. Modelling shows greater reproduction rates in urban areas [48,76], and epidemiological data are skewed towards higher cases in urban areas across all countries [54,77]. A similar pattern was observed for Ebola [78]. South Africa, clearly the COVID-19 exception in the continent, is a rapidly urbanizing outlier, as only 30% of its population live in rural areas [75]. Nigeria, on the other hand, models the trends seen in the continent as a whole, with approximately a 50% rural population [75] and a low COVID-19 burden [1]. Limited research in Africa also shows significantly more intergenerational contacts in rural as compared to urban areas [79]. Researchers posit this distribution, household size, and patterns of age-structured social contacts modify the spread of epidemics [48,76,79,80]. Furthermore, a recent study depicts the effect that the nature of social networks and mobility have on COVID-19 transmission [81]. Communities with increased social capital tend to see worse disease outbreaks overall [81,82], although this is not always the case [83]. An increased feeling of integration and connection to society is beneficial in terms of social support, which could potentially benefit individual health outcomes, but may generally have negative consequences for containing infectious disease spread due to increased human contact [80].

The limited research conducted on social contact in Sub-Saharan Africa (SSA) shows that enhancing social distance mitigation strategies, particularly for elderly populations, would result in mortality decreases, but not to the extent that these changes would have in higher income settings, which tend to have increased proportions of elderly cohorts in the population [9]. The small slice of the African population who are older (only 3% of the African population is 65+) [3] live overwhelmingly at home, often with extended families spanning multiple generations. This alone explains a huge discrepancy in cases, as roughly one third to one half of deaths in wealthy countries, such as the U.S., have resulted from superspreading events in elderly nursing homes and assisted living facilities, providing the rationale for prioritizing the inoculation of these older individuals [84,85]. While multiple family homes generally have more people in a shared space than the typical single-family homes of Western countries, this slightly increased risk of within-household spread is offset by the significantly decreased risk of large-scale superspreading events in the community, often caused by congregate nursing home settings [86]. Despite an increasing trend of elders being cared for in long-term care facilities in Africa [87], especially in South Africa [88], this is still far less commonly practiced than in Western countries, Asia [89], or Latin America [90,91].

### 2.3. A Largely Outdoor Existence 

Because infected persons transmit the virus through coughing, sneezing, talking, singing, and breathing [92], living environments matter. Further, viability and infectivity are influenced by environmental conditions [46,93]. Studies show that coronavirus transmission, while also possible outdoors [94], is concentrated in indoor settings where it is estimated to be about 19 times higher [95]. While most likely only minimally contributing to viral spread, built environments requiring ventilation, air-conditioning/heating, wastewater, and sewer systems have been shown to carry the virus that may escape through aerosolization [96,97,98]. These systems are generally in urban areas and are almost entirely lacking in rural Africa where most people live. In contrast, respondents in the U.S. National Human Activity Pattern Survey reported spending over 90% of time in enclosed environments, either in buildings or in their cars [96,99]. 

African livelihoods that are largely dependent on agriculture and pastoralism favor dawn-to-dusk outdoor lifestyles, with shelters used mostly for sleeping. Research shows people in rural areas spend far more time outdoors as compared to urban areas [100]. Even in the case of sleeping, these homes are often well ventilated with outside air, significantly reducing the chance of viral transmission when compared to tightly enclosed indoor spaces in developed countries. Additionally, higher temperatures and UV light intensity have been shown to predict SARS-CoV-2 spread [101,102], although the evidence is inconsistent [103]. Prolonged, year-round outdoor living with direct exposure to UV light in mostly warm and tropical climates could partially explain reduced transmission [46], perhaps due to endogenously produced vitamin D, which is suggested in some studies, including a systematic review and meta-analysis, to attenuate COVID-19 symptom severity [104,105]. Vitamin D supplements are under several ongoing clinical investigations [106]. 

As a final note regarding the extent of transmission, even if the SARS-CoV-2 virus is more widespread than reported as seroprevalence data suggest, there still has been much less morbidity and mortality observed. The proposed factors reviewed above provide some insight into how African-specific transmission patterns have emerged and evolved over time.

## 3. Factors Mitigating COVID-19 Burden in Africa

Even though case and death reporting has certainly been less reliable in Africa, there has been very little evidence of increased overall mortality or widespread COVID-19 disease, with the exceptions of South Africa and northern African countries. We now examine what could possibly explain this somewhat perplexing situation.

### 3.1. Demographic Pyramid

**Figure 1:**
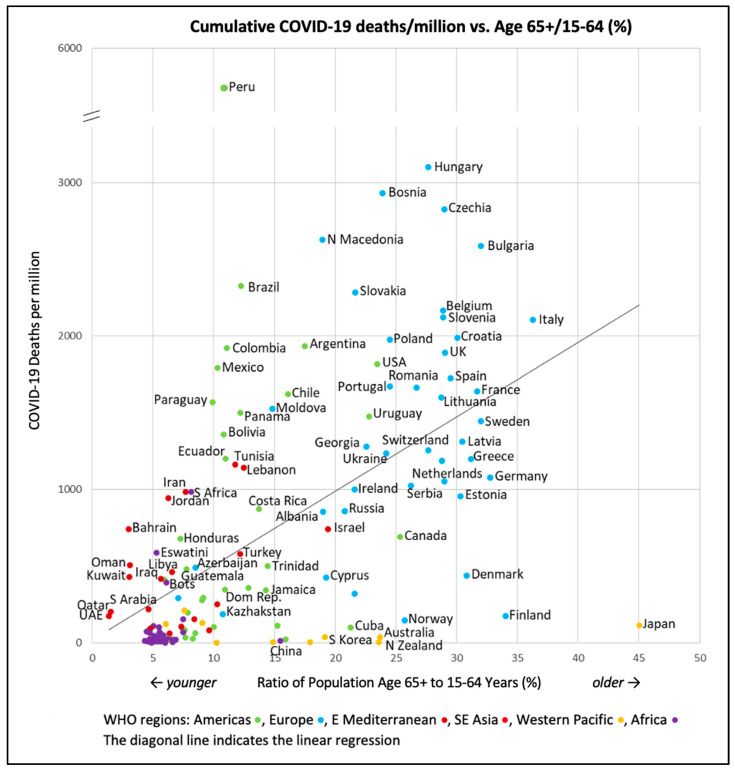
This analysis is based on data extracted from ourworldindata.org accessed on 17 June 2021, retaining data on countries with populations of at least 1 million, for which complete data were available for the analyses done. Among these countries, as one would expect, COVID-19 mortality is strongly correlated with age structure. Note the concentration of purple dots in the bottom left, indicating comparatively low COVID-19 mortality and young age structure among most African countries. The Pearson’s R^2^ for cumulative COVID-19 mortality and the ratio of persons aged 65+ to those aged 15–64 is 0.283; for example, 28.3% of the between-country variance in cumulative COVID-19 mortality can be accounted for by age structure alone.

It is beyond doubt that the demographic pyramid is significantly related to decreased COVID-19 burden. It is well documented that COVID-19 burden is heavily skewed towards older populations [107,108], as demonstrated by a study of 17 million COVID-19 cases [109]. Compared with a reference demographic group of 5–17 years, the demographic of 65–74 years is 35 times more likely to become hospitalized from SARS-CoV-2 infection, and 1100 times more likely to die from COVID-19, with these risks increasing significantly in even higher age groups [108,109]. Africa has the youngest population among all global regions, with a median age of 19.7 years [25,51]. Conversely, the median ages among the hardest hit countries are much higher: 26.8 years in India [110], 31.4 years in Brazil [111], 38.5 years in the U.S. [112], and 40.5 years in the U.K. [113]. Modelling clearly shows that the COVID-19 mortality for Africa tracks this similar age pattern [48,54], and this is confirmed by actual current mortality data [114]. Through conducting a simple linear regression to show between-region differences, regressing cumulative mortality per 1 million population on the ratio of population aged 65+ vs. aged 15–64, the R^2^ = 0.283 is found, meaning that the variance in mortality accounted for by age structure is 28.3% (Figure 1) [2,75,115]; the cumulative mortality figures used here are for the period since the beginning of the pandemic through 17 June 2021, as reported on oneworldindata.org (accessed on 17 June 2021), which utilizes Johns Hopkins University Centers for Systems Science and Engineering COVID-19 data [2]. These data provide evidence that despite the considerable spread of infection, COVID-19 disease and mortality burden in younger African populations is comparatively absent. However, South Africa shows a much higher mortality than many countries with a similar age structure, including India and Egypt, meaning that other factors are also at play.

### 3.2. Pre-Existing Conditions

It is well known that people with pre-existing conditions, such as diabetes, chronic respiratory diseases, obesity, and hypertension have a greatly increased risk of moderate to severe complications from COVID-19 infection [53,116,117]. Broadly, these conditions are considerably less prevalent in low income and lower middle income countries (LICs and LMICs) when compared to higher income countries (HICs) [9,118], providing an additional possible explanation for why COVID-19 burden is more reduced in the African continent. Indeed, African countries have a low prevalence of NCDs (only accounting for 29.8% of total burden of disease in SSA, with the majority of burden coming from infectious disease) [119], compared to 88% in the US and 74% in Brazil [120], which align with the impact of pre-existing conditions on increasingly severe complications and death from COVID-19 [9,116]. South Africa, which accounts for nearly 40% of all reported COVID-19 cases and deaths in the continent [2], reports an exceptionally high burden of NCDs [119,121]. However, some research suggests that the prevalence of infectious disease can similarly exacerbate COVID-19 burden and may actually indicate that regions with high infectious disease and low NCD prevalence (such as in Africa) are not advantaged [9,25]. For instance, a recent cohort study in South Africa suggested that HIV was associated with a doubling of mortality risk of COVID-19 [122]. This is potentially significant to consider in explaining why South Africa has a disproportionate COVID-19 burden in the continent, given that it also has the greatest number of people living with HIV/AIDS in the world [123]. More studies are needed, however, before this potential association can be determined.

### 3.3. Trained Immunity

The phenomenon of trained immunity may be tempering the COVID-19 burden in the continent. Here, we focus on four elements underlying this hypothesis: (i) BCG vaccinations, (ii) exposure to varied commensal microorganisms, or the “hygiene hypothesis”, (iii) prevalence of infectious diseases, and (iv) historical use of herbal plants and remedies.

(i) Live vaccines activate innate immune systems, conferring protection against future infections from other pathogens [124,125,126,127,128,129], which researchers believe may have the potential to also attenuate consequences of infection with SARS-CoV-2 [130]. Recent data suggest that regions with mandated BCG vaccinations have had lower COVID-19 disease burden [131], which may speak to an association between BCG vaccination rate and population COVID-19 burden. Children vaccinated with BCG could have a lower infection risk with SARS-CoV-2 [132], continuing well into adulthood. Interestingly, and applicable to COVID-19, BCG vaccination was shown to be especially protective against complications of other respiratory viral infections, supported by studies in Guinea-Bissau and South Africa [133,134]. Additionally, in rodent models, BCG reduces viral load from infection by influenza A and herpes simplex virus type 2 (HSV2) [135,136], with a mediation by a boosted innate, nonspecific immune defense via increased cytokine production and macrophage action. It is not known if BCG immunity confers such protection in older populations [132], but this has been suggested by some research [137]. This is hypothesized in part from observations in countries that lagged behind other efforts to disseminate BCG, such as Iran and Somalia, which have incurred a significant death toll from COVID-19. Still, it is possible that countries with earlier BCG administration campaigns have contributed to the protection of older populations from heavy COVID-19 burden, through childhood inoculation for tuberculosis [137]. Because COVID-19 complications are often a result of significant systemic inflammation [138,139], the fact that inoculation with BCG boosts an innate immunity that subsequently lowers the extent of inflammation [132] indicates that it could be a pathway by which BCG attenuates infection of SARS-CoV-2. Of course, controlled clinical trials are needed to verify this hypothesis. While this varies, most African countries have high BCG coverage [140], with the notable exception of Somalia, due to the long-standing civil wars interfering with child vaccination programs. As noted in one study, countries without universal policies for BCG vaccination (such as Italy, the U.K., Spain, and the U.S.) have experienced much more severe disease burden compared to countries with universal programs (including most African countries and Japan, for example) [137]. However, this association may be primarily due to other factors; for example, countries that have a BCG vaccine mandate may tend to have stricter public health measures in place that could indirectly affect population COVID-19 burden, instead of the effect being driven primarily by BCG vaccination. Furthermore, if this BCG hypothesis turns out to have some merit, countries in Africa with similar levels of BCG coverage and population structure should show comparable COVID-19 burden.

(ii) The so-called “hygiene hypothesis” posits that some environments advantage populations against certain forms of infection and disease, due to chronic exposure to a multi-microbial environment, potentially producing protective immune effects when encountering new pathogens [130,141]. There has been some concern regarding regions that use ultra-hygienic practices, exemplified by the overuse of hand sanitizer and other disinfection practices in many countries, as inadvertently creating a disadvantage for confronting new immune challenges such as SARS-CoV-2 [130]. Accordingly, non-specific immunity would be weakened and may have implications for disrupting the adaptive composition of commensal organisms on the skin, gastrointestinal tract, and other organ systems [130]. Because COVID-19 is a relatively new viral problem, it may take a while before conclusive statements can be made about the role of the microbial environment on infection susceptibility, but researchers agree that this hypothesis is plausible [130], in part indicated by the aforementioned dichotomy of burden between richer and poorer countries [142,143]. Higher income countries (with a few exceptions) have suffered much greater COVID-19 burdens (specifically, hospitalization and death rates) than the poorest countries, on average [2]. Such data raise the consideration that richer countries could be maladaptively over-sanitizing. 

(iii) Given that 22 of the 25 most vulnerable countries to infectious disease epidemics are in Africa (the other three being Afghanistan, Haiti, and Yemen) [144], the continent carries the heaviest burden of infectious diseases, including the impoverishing neglected tropical diseases (NTDs) [145,146]. During 2018 alone, SSA faced 96 disease outbreaks in 36 of 47 countries [55]. This pathogenic environment precipitates the wide use of antibiotics, antimalarials, and other drugs to treat NTDs, such as azithromycin and ivermectin often distributed through mass drug administrations [147,148,149,150,151], which might counteract to mitigate COVID-19 morbidity. In particular, used widely over several decades in SSA, ivermectin has been spotlighted as a potential treatment for COVID-19 [152,153], including by the NIH [154,155]. Researchers have postulated that “circulating viruses or parasites in the African subcontinent” could explain high SARS-CoV-2 antibody seropositivity [14]. For instance, of 228 million cases of malaria worldwide in 2018, 93% were in SSA [156]. Notably, South Africa is not generally endemic for malaria and other NTDs [157]. Intense malaria exposure (which is frequent in many rural areas in SSA and much less so in urban areas, and not at all in South Africa or in northern Africa countries) has a strong influence on the immune system and could contribute to a better trained immunity [158,159]. It is possible that infection by malaria alone may overstimulate the immune system and confer an immune advantage when compared to nonexposed populations. To further investigate this potential role, as very few to no communities outside of Africa are holo-endemic for the disease [160], mechanistic studies would be needed to determine if there is cross-immunity between malaria and SARS-CoV-2 exposure.

(iv) Yet to be measured, the historical use of natural medicine for primary care [161,162,163] and widespread belief of self-medication with these for COVID-19 in Africa has triggered a WHO-AFRO expert panel in September 2020 to endorse a protocol for the clinical investigation of herbal medicine for COVID-19 [164]. During 30 March–1 April 2021, the Third Regional Consultation with Experts and Researchers on the Contribution of Traditional Medicine to COVID-19 Response in the African Region was held, with contributions from scores of scientists and countries. The case for exploring natural medicine in the fight against COVID-19 is justified [165]. Although African countries such as Madagascar have endorsed the wide use of a traditional therapeutic agent to fight COVID-19, there are no published scientific data that would lend support to this claim. Clinicaltrials.gov (accessed on 7 July 2021) reports that several studies are underway, including Chinese traditional herbal medicines [166]. The establishment in Africa of a Regional Expert Advisory Committee on Traditional Medicine for COVID-19 compromising 25 members speaks to the widespread use of and belief in herbal medicine as possible means of prevention or cure of COVID-19.

### 3.4. Genetics

Recent work suggests that there is a role for genetics in COVID-19 trajectory, and in differentially affecting separate populations [167,168]. Some genetic immunological factors could possibly be playing a role in shielding Africa from the brunt of the pandemic [41]. For example, SARS-CoV-2 infects human cells largely through its interactions with the ACE2 receptor, involved in regulating blood pressure dynamics [6,117,169]. Populations varying in the expression of the ACE2 protein may have different baseline ‘openness’ for infection. African people have been shown to respond less effectively to ACE inhibitors for treatment of blood pressure, and have less expression of ACE2; therefore, there is potential for a more difficult route that the virus must maneuver to infect cells in this population [6,169]. There also may be genetic susceptibility via the 3p21.31 gene cluster, as one GWAS study showed ABO blood group A as having the highest risk of COVID-19-associated respiratory failure, with group O having the lowest risk [170]. Studies have shown that African populations have a particularly high proportion of O-positivity at nearly 50%, which is higher than in White and Asian populations [171,172,173]. It is possible that this increased O prevalence could be conferring a greater protective effect in African populations compared with other groups with less O prevalence; however, no studies have concluded this. While this hypothesis is somewhat challenged by the particularly heavy COVID-19 burden facing African Americans in the U.S. [174], who would likely share some or most of these genetic advantages [175,176], elevated levels of NCDs are observed more in African Americans than in continental African populations [119,177,178], which could help explain this discrepancy along with other adverse socioeconomic and cultural factors.

### 3.5. Broader Sociocultural Implications

Importantly, inequality of distribution of income (standardized by the GINI co-efficient) appears to be at least partially correlated with increasing disease burden of COVID-19, as South Africa (with a value of 63.1, among the highest in the world) and Brazil (54.7) [179] have been hit hard by COVID-19. One study of the top 50 countries with the highest numbers of cases suggests that increased income inequality was associated with increased severe cases and mortality [53]. Across the US, Brazil, South Africa, and Europe, increased mortality has been reported among minority groups such as Africans and Asians [180,181]. Three countries (South Africa, Brazil, the U.S.) seem to have similar risk profiles: long historical cleavages of institutional racism and inequality [182,183] that exacerbate COVID-19 vulnerabilities among large Black populations. The role in which inequality and poverty play totally depends on the behavior and the biological/immunological factors that they influence. For example, in New York and in other U.S. cities, poor people have difficulty isolating themselves, as they commonly utilize public transportation, share living spaces with more people, work in crowded environments, and overall, have lower social mobility [184]. In contrast, in many African cities, the social and political elites are the ones who can afford to live and work in airconditioned closed spaces, increasing susceptibility to infection through close, indoor contact with others [185]. However, this should not be generalized as the case in every African country, as work has also pointed to the similar theme of poorer populations facing a higher burden, such as in South Africa, with the elites largely shielded from the virus [186].

## 4. Conclusions

Relatively low severity and death due to COVID-19 in Africa presents somewhat of a paradox [41,42,187]. Despite early ‘doomsday’ predictions for Africa, the continent succeeded in stemming the first wave of SARS-CoV-2 spread [188], although the second wave was more severe [21]. On the whole, the factors discussed here have contributed to modulating disease spread and severity; however, the strength of evidence of each varies. A third wave that is currently underway in many countries appears to bring more consequential morbidity and mortality concerns, and possible impacts [189], especially as early government measures have been relaxed in many countries [21]. Driven by more virulent and potentially lethal variants, such as Delta, this third wave may prove far more challenging for weaker health systems to cope with, leading to increased hospitalizations and deaths [190]. Future waves are also likely. Alongside facing the lowest quality of health systems [9,191], the continent will be significantly challenged if it faces an excessive COVID-19 disease burden [192]. Estimates already show that many excess deaths, especially in the SSA region, will result not from COVID-19 but from disruptions in programs addressing malnutrition [193], HIV/AIDS [194], malaria [195], and maternal and child health deaths [32], along with interruptions in the implementation of immunization programs [196]. Therefore, a range of policy options taking this into account, as well as considering economic and socio-cultural characteristics including the expected benefits and harms of control measures (e.g., adverse effects on education and livelihoods) [7,42], need to be implemented.

It is likely that SARS-CoV-2 has already been widely disseminated through Africa, yet evidently without having had the severe consequences of COVID-19 burden, such as the significant uptick in hospitalizations and deaths that many other regions have experienced [25]. If so, widespread infection is likely to also result in widespread natural immunity [197]. While the true picture of infections and mortality in the continent has yet to fully emerge, the quality of data for other diseases, such as HIV/AIDS, indicates that Africa has the capacity to collect and report valid disease surveillance data [198], which should give a degree of confidence in the existing COVID-19 data and the ability for the continent to do better. Nevertheless, improving completeness of data collection and reporting is an ongoing mission for Africa and elsewhere [30,199]. Strengthening lab capacities, validating current rapid tests in the context of other infectious diseases, and standardizing data and survey reporting will also expand the true picture [42]. Additionally, before the COVID-19 pandemic in 2019, researchers and policy makers from Africa called for a new model of public health in the 21st Century, precisely to prepare for this kind of pandemic [200]. Now, as the pandemic evolves, the vaccine rollout needs to be especially accelerated to reach more people, as the continent lags with less than 5% of its population having received at least one shot as of August 2021 [201]. We also urge the continuation of measures for which there is clear evidence of effectiveness [202]. While what has been observed is a comparatively low morbidity and mortality from COVID-19 in Africa, the continent faces a significant threat with the current progression of the pandemic that may change what has been seen thus far [203]. Still, as our assessment here shows, the unique experience of African countries may offer salient lessons for the rest of the world, given their long experience with infectious diseases and outbreak readiness [56,57].

## Data Availability

Not applicable.
